# Real-world outcomes of sitagliptin and sitagliptin/metformin single-pill combination treatment in diverse type 2 diabetes populations (DIVERSITY study)

**DOI:** 10.3389/fcdhc.2026.1905393

**Published:** 2026-08-26

**Authors:** Andrej Janez, Mirjana Bojic, Tomislav Bozek, Zdravko Kamenov, Tatjana Milenkovic, Gabriela Radulian, Tamás Várkonyi

**Affiliations:** 1 Department of Endocrinology, Diabetes and Metabolic Diseases, University Medical Centre Ljubljana, Ljubljana, Slovenia; 2 Department of Endocrinology, University Clinical Center of the Republic of Srpska, Banja Luka, Bosnia and Herzegovina; 3 Vuk Vrhovac University Clinic, Clinical Hospital Merkur, Zagreb, Croatia University of Zagreb School of Medicine, Zagreb, Croatia; 4 Clinic of Endocrinology, Alexandrovska University Hospital, Sofia, Bulgaria; 5 University Clinic of Endocrinology, Diabetes and Metabolic Diseases, Skopje, North Macedonia; 6 National Institute of Diabetes, Nutrition and Metabolic Diseases N. Paulescu, Bucharest, Romania; 7 Department of Internal Medicine, Albert Szent-Györgyi Clinical Center, Szeged, Hungary

**Keywords:** DPP-4 inhibitor, HbA1c, hypoglycemia, real-world evidence, safety, sitagliptin, sitagliptin/metformin, type 2 diabetes

## Abstract

**Introduction:**

Real-world evidence on dipeptidyl peptidase-4 (DPP-4) inhibitors is limited by selective populations in randomized trials. The international, prospective DIVERSITY study evaluated the effectiveness, safety, and treatment acceptability of sitagliptin and sitagliptin/metformin single-pill combination (SPC) in routine clinical practice across diverse type 2 diabetes (T2D) populations.

**Methods:**

This non-interventional, multicenter study enrolled adults with T2D eligible for sitagliptin or sitagliptin/metformin SPC and followed them for 6 months with three data captures (baseline, ~3 months, ~6 months). The analysis set comprised 2,603 patients (mean age 64.6y ± 10.6; 53.1% women) meeting all criteria and with ≥152 days between first and third captures. Primary outcomes were absolute changes in glycated hemoglobin (HbA1c), fasting plasma glucose (FPG), and post-prandial glucose (PPG) from baseline to 6 months. Secondary outcomes included the proportion achieving HbA1c <7% at 3 and 6 months, changes in body mass index (BMI), and hypoglycemia incidence. Safety was assessed in all treated patients (safety analysis set [SAS], n=2,697).

**Results:**

Glycemic control improved over the 6-month observation period. Among patients with paired data, mean HbA1c fell from 7.98% to 6.99% (Δ −0.99%, 95% CI −1.05 to −0.93). Reductions were observed with both regimens: sitagliptin monotherapy (Δ −0.93%, 95% CI −1.01 to −0.85) and sitagliptin/metformin SPC (Δ −1.03%, 95% CI −1.11 to −0.96). FPG decreased by −1.91 mmol/L (95% CI −2.03 to −1.79) and PPG by −2.39 mmol/L (95% CI −2.56 to −2.21). The share of patients with HbA1c <7% rose from 16.9% at baseline to 53.7% at 6 months. BMI changes were small and consistent with weight neutrality (mean −0.61 ± 1.33 kg/m²). Treatment-related symptoms of hypoglycemia were rare (3 cases [0.1%] of all treated patients) and treatment acceptability was high: ≥90% patients and investigators reported satisfaction at 6 months.

**Conclusions:**

In routine practice across heterogeneous T2D populations, sitagliptin and sitagliptin/metformin SPC were associated with clinically meaningful improvements in HbA1c, FPG, and PPG over 6 months, with effects consistent with a weight-neutral profile, a low reported incidence of hypoglycemia, and high treatment satisfaction. These findings are consistent with the use of sitagliptin-based regimens, whether used as monotherapy or as add-on therapy, as treatment options in routine real-world care, including in older and multimorbid patients.

## 1 Introduction

Diabetes mellitus represents one of the most significant global health challenges, with recent estimates indicating that 10.5% of adults worldwide are affected, a prevalence driven by aging populations, urbanization, and lifestyle changes (1, 2). Type 2 diabetes (T2D) accounts for more than 90% of all cases globally (2). Beyond its rising prevalence, diabetes is strongly associated with multiple comorbidities, including cardiovascular disease, chronic kidney disease, neuropathy, and retinopathy. Evidence suggests that individuals who develop T2D often present with cardiometabolic comorbidities, such as hypertension, dyslipidemia, and non-alcoholic fatty liver disease, long before diagnosis (3).

Despite advances in therapy, nearly 50% of patients fail to achieve recommended glycemic targets (4). Given the chronic and progressive nature of T2D, most patients eventually require dual or triple therapy. Current guidelines advocate a stepwise approach, intensifying treatment at 3-month intervals until individualized glycemic goals are met. Importantly, therapeutic intensification typically involves adding agents rather than replacing them (5).

The 2022 ADA/EASD consensus report recommends tailoring treatment according to cardiovascular risk and positions gliptins as an add-on therapy option to metformin and/or sodium–glucose cotransporter 2 (SGLT2) inhibitors (5). Sitagliptin is of particular interest due to its advantages: weight neutrality, low incidence of adverse events, simple dosing, few contraindications and suitability for patients with hepatic impairment (6, 7).

Previous studies have evaluated the efficacy and safety of sitagliptin, but most were conducted under controlled conditions with narrowly defined patient populations (8). The international DIVERSITY study was designed to address this gap by assessing the use of sitagliptin and sitagliptin/metformin SPC in routine clinical practice, where physicians treat heterogeneous patients with varying risk profiles and treatment regimens. The primary objective was to generate real-world evidence on the effectiveness and safety of sitagliptin and the sitagliptin/metformin single-pill combination across diverse patient subgroups. Additionally, the study aimed to characterize patterns of comorbidities, associated risk factors, and diabetes-related complications under different therapeutic strategies.

## 2 Methods

### 2.1 Study design

The DIVERSITY study was an international (Bosnia and Herzegovina, Bulgaria, Croatia, Hungary, North Macedonia, Romania and Slovenia), non-interventional, observational, prospective multicenter study designed to evaluate the effectiveness and safety of sitagliptin and sitagliptin/metformin SPC in various groups of T2D patients. Between January 2023 and June 2025, a total of 2697 adult patients with T2D were investigated.

The study involved 206 investigators, including diabetologists, endocrinologists and other specialists routinely treating type 2 diabetes. Prior to enrollment, all patients received comprehensive verbal explanations of the study procedures from the investigators, presented in clear and understandable language. Additionally, patients were provided with written information in their native language.

Data collection was conducted exclusively on patients who provided informed consent for the use, analysis, and disclosure of their personal data in compliance with the European General Data Protection Regulation (GDPR, Regulation 2016/679). The study was carried out in accordance with the ethical standards of the Declaration of Helsinki, good pharmacovigilance practice and the applicable national legislation governing epidemiological research in each participating country. The study did not interfere with the standard care practice, and any follow-up procedures were not part of this study. The study protocol was reviewed and approved by the Ethics Committees of all participating countries, with documented approvals obtained in accordance with local regulations.

### 2.2 Eligibility criteria

Adults (≥18 years) with T2D eligible for sitagliptin or sitagliptin/metformin SPC were enrolled, including patients inadequately controlled on metformin, patients uncontrolled on diet and exercise with metformin contraindications or intolerance, patients requiring treatment intensification beyond metformin plus another oral antidiabetic agent (sulfonylureas, glinides, sodium–glucose cotransporter-2 inhibitors, meglitinides, glucagon-like peptide-1 receptor agonists, thiazolidinediones), and patients with insufficient glycemic control on stable insulin therapy with or without metformin.

Key exclusion criteria presented inability to follow clinical practice for any reasons, participation in other clinical study and contraindications listed in the sitagliptin, sitagliptin/metformin SPC prescribing information.

### 2.3 Study medication

At baseline visit, all enrolled patients were prescribed either add-on therapy or newly initiated therapy with generic sitagliptin Maysiglu^®^ or sitagliptin/metformin SPC Maymetsi^®^ (produced by Krka, d. d., Novo mesto, Slovenia).

### 2.4 Data collection

Patients were prospectively followed for 6 months, with data collected at baseline, 3 months, and 6 months. All visits were conducted according to routine clinical practice in each participating country.

At baseline, investigators recorded patient demographics, body weight and height, smoking status, and laboratory values (glycated hemoglobin (HbA1c), fasting plasma glucose (FPG), and postprandial glucose (PPG)). The prescribed dose of sitagliptin or sitagliptin/metformin SPC, detailed medical history (including date of type 2 diabetes diagnosis), concomitant diseases, previous antidiabetic therapy and relevant risk factors were also documented. At the 3-month visit, body weight, height, HbA1c, ongoing dose of sitagliptin or sitagliptin/metformin SPC, and any changes in antidiabetic or concomitant treatments were collected, along with safety assessments. At 6 months, investigators recorded body weight and height measures, laboratory parameters (HbA1c, FPG, PPG), treatment changes, continued dosing and both patient and investigator satisfaction with the therapy.

### 2.5 Aims and outcomes

The primary objective of the DIVERSITY study was to evaluate the effectiveness and safety of sitagliptin and sitagliptin/metformin SPC in routine clinical practice across patients with diverse clinical characteristics, risk profiles, and treatment-based subgroups.

The primary outcomes were the absolute changes in HbA1c, FPG and PPG from baseline to 6 months (± 1 month). Secondary outcomes included the proportion of patients achieving HbA1c <7% at 3 and 6 months (± 1 month), absolute and relative changes in BMI between baseline and 6 months, and the incidence of hypoglycemia at the 3- and 6-month assessments. Exploratory outcomes encompassed characterization of antidiabetic therapy prior to baseline, evaluation of concomitant antidiabetic treatment at each visit, reasons for modifying sitagliptin or sitagliptin/metformin therapy, and patient and investigator satisfaction and patient-reported response after 6 months. Safety analyses focused on treatment-emergent adverse reactions recorded at each follow-up visit.

### 2.6 Statistical analysis

Each endpoint (primary and secondary) was assessed by reporting the appropriate summary statistics.

Summary statistics consists of the number of patients/observations, frequencies and corresponding percentages for categorical variables, and of the number of patients/observations, mean, median, standard deviation, minimum and maximum, first and third quartile for numeric variables.

Some endpoints (in particular, the primary endpoint) were assessed by the appropriate methods of inferential statistics. The inferential methods pertain to the assessments of proportions and the assessments of expected values of numeric variables (means and mean differences). For inferential assessment of proportions, the two-sided “equal-tails” Clopper-Pearson exact 95%-confidence interval was reported. For the assessment of means and mean differences, the standard 95%-confidence interval for the mean (with unknown variance) based on the limiting distribution of the standardized sample mean was reported (together with the corresponding p-value). For the purpose of the article, additional analyses were performed to further support interpretation of the main study findings. These analyses included an adjusted assessment of change in HbA1c from baseline to the last follow-up visit, accounting for baseline HbA1c and relevant clinical characteristics, and an evaluation of baseline HbA1c values in patients with and without available follow-up HbA1c measurements. The aim of these analyses was to assess the robustness of the observed HbA1c changes and to explore the potential impact of missing follow-up data on the interpretation of treatment outcomes.

## 3 Results

Data from 2,697 patients were collected during the study and comprised the Safety Analysis Set (SAS). The Full Analysis Set (FAS) included 2,603 patients. Overall, 94 patients (3.5% of the SAS) were excluded from the FAS because they did not meet one or more predefined eligibility criteria. The reasons for exclusion were an insufficient interval between the first and third data collection visits (n = 62, 66.0%), a missing date of the third data collection visit (n = 30, 31.9%), and incomplete data collection (n = 14, 14.9%). Patients could be excluded for more than one reason; therefore, the frequencies and percentages across exclusion categories do not sum to the total number of excluded patients.

### 3.1 Baseline demographic and clinical characteristics

Mean age of patients was 64.6 ± 10.6 years, with women comprising 53.1% of the total cohort. Average duration since T2D diagnosis among patients was 8.6 years (8.68 ± 7.29 years) and the average BMI was 29.70 ± 4.64 kg/m^2^. Most of the patients (68.9%) were non-smokers.

**Figure 1:**
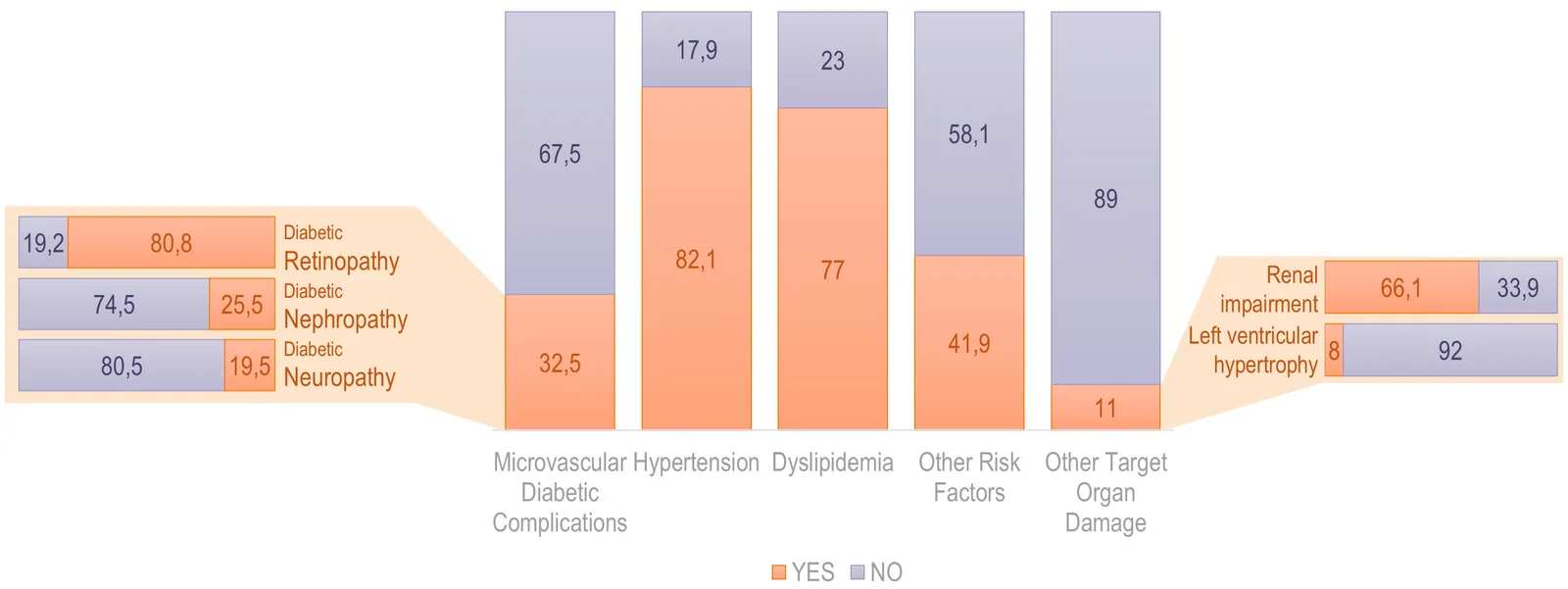
Concomitant diseases and risk factors.

At baseline, 87.6% of patients presented with at least one concomitant disease, presented in **Figure 1**. The most prevalent comorbidities were arterial hypertension (82.1%) and dyslipidemia (77%). Additional conditions included hypothyroidism, hyperuricemia, myocardial ischemia, atrial fibrillation, and other cardiovascular or metabolic disorders. Most common diabetes-associated conditions were microvascular diabetic complications (32.5%). Among those affected by microvascular diabetic complications 80.5% were diagnosed with diabetic neuropathy, 74.5% with diabetic nephropathy and 19.2% with diabetic retinopathy. Only 11.0% of patients had other target organ damage at the start of treatment, of whom 66.1% presented with left ventricular hypertrophy and 8.0% had renal impairment (defined as eGFR <30 mL/min/1.73 m²). Concomitant pharmacotherapy was highly heterogeneous, comprising approximately 262 distinct International Nonproprietary Names (INNs).

Majority of patients, 94.6%, were previously treated with T2D medications. Most used were metformin in 71.8% patients, gliclazide in 20.8% patients, long-acting insulin in 7.8% patients, empagliflozin in 7.4% patients and sitagliptin/metformin SPC in 7.3% patients.

### 3.2 Treatment regimen

**Figure 2:**
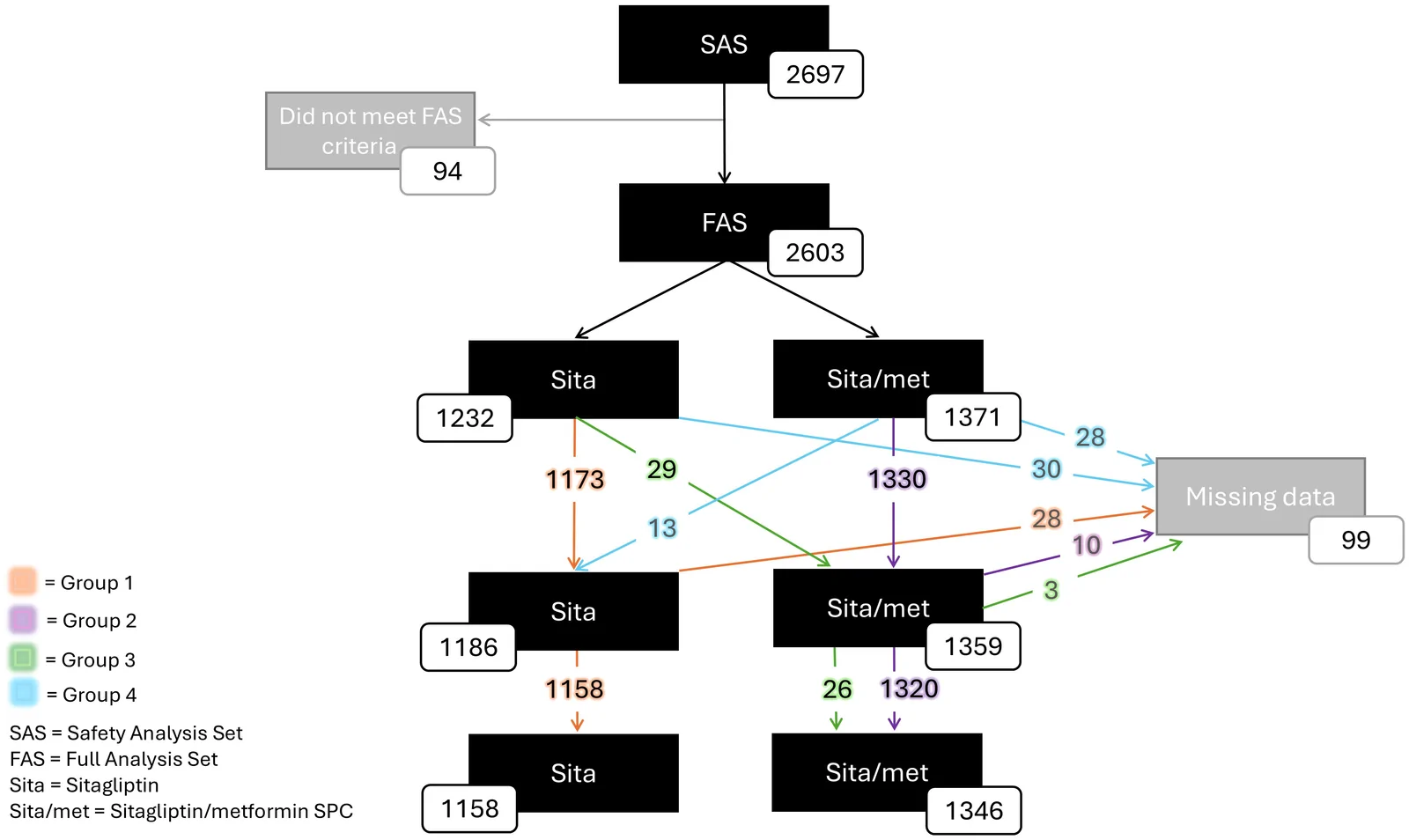
Patient disposition and treatment pathways during the study.

A total of 2,603 patients were initially assigned to one of two sitagliptin-based treatment regimens: sitagliptin (47.3%, n = 1,232) or sitagliptin/metformin single-pill combination (SPC) (52.7%, n = 1,371). For the analyses presented in this report, patients were classified according to treatment received at Visit 1 and Visit 2. Group 1 included patients who received sitagliptin at both visits (n = 1,173), Group 2 included patients who received sitagliptin/metformin SPC at both visits (n = 1,330), and Group 3 included patients who received sitagliptin at Visit 1 and switched to sitagliptin/metformin SPC by Visit 2 (n = 29). Patients who did not follow any of these treatment pathways were categorized as Group 4 (n = 71). As the aim of this report was to evaluate outcomes associated with the predefined sitagliptin-based treatment patterns, results for Group 4 are not presented. Treatment modifications during follow-up were made at the discretion of the treating physician because of inadequate glycemic control, safety considerations, contraindications, or other clinical reasons. Detailed treatment pathways and subgroup definitions are presented in **Tables 1** and **2** and **Figure 2**. The most prescribed doses were sitagliptin 100 mg (44.4% of patients at Capture 1 and 43.6% at Capture 3) and sitagliptin/metformin SPC 100 mg/2,000 mg (46.0% and 47.8%, respectively). Group 2 was further divided into subgroups 2a-2d according to the background glucose-lowering therapy received alongside the sitagliptin/metformin SPC (SPC without further glucose-lowering therapy, SPC with a sulfonylurea, SPC with an SGLT2 inhibitor, and SPC with insulin). These four combinations were selected because they represent the most frequent prescribing patterns in routine care in the participating countries and differ in baseline glycemic burden and in hypoglycemia risk, so that reporting them separately avoids masking heterogeneity within Group 2.

**Table 1:** Definition of treatment groups based on sitagliptin-based therapy received at Visits 1, 2, and 3.

Treatment group	Visit 1		Visit 2		Visit 3	n
Group 1	Sita	→	Sita	→	Sita	1173
					Sita + other*	
	Sita + other*		Sita + other*		Sita + met + other**	
					SPC sita/met + other**	
Group 2	SPC sita/met	→	SPC sita/met	→	SPC sita/met	1330
					SPC sita/met + other**	
	SPC sita/met + other**		SPC sita/met + other**		Sita	
					Sita + other*	
Group 3	Sita	→	SPC sita/met	→	SPC sita/met	29
					SPC sita/met + other**	
	Sita + other*		SPC sita/met + other**		Sita	
					Sita + other*	

*other antidiabetics (insulin, SGLT2i, SU, TZD), including metformin.

**other antidiabetics (insulin, SGLT2i, SU, TZD), but not metformin.

**Table 2:** Definition of group 2 subgroups according to concomitant glucose-lowering therapy.

Treatment group	Visit 1		Visit 2		Visit 3	n
Group 2a	SPC sita/met	→	SPC sita/met	→	SPC sita/met	775
					SPC sita/met + other**	
	SPC sita/met + other***		SPC sita/met + other***		Sita	
					Sita + other*	
Group 2b	SPC sita/met + SU	→	SPC sita/met + SU	→	SPC sita/met + SU	276
					SPC sita/met + SU+ other**	
			SPC sita/met + SU + other***		Sita	
					Sita + other*	
Group 2c	SPC sita/met + SGLT-2i	→	SPC sita/met + SGLT-2i	→	SPC sita/met + SGLT-2i	156
					SPC sita/met + SGLT-2i + other**	
			SPC sita/met + SGLT-2i + other***		Sita	
					Sita + other*	
Group 2d	SPC sita/met + insulin	→	SPC sita/met + insulin	→	SPC sita/met + insulin	123
					SPC sita/met + insulin + other**	
			SPC sita/met + insulin + other***		Sita	
					Sita + other*	

*Other antidiabetics (insulin, SGLT2i, SU, TZD), including metformin.

**Other antidiabetics (insulin, SGLT2i, SU, TZD), but not metformin.

***Other antidiabetics (BG, DPP-4, DPP-4/BG, DPP-4/TZD, GLP-1, SGLT2i/BG, SGLT2i/DPP-4, SU/BG, SU/TZD, TZD, TZD/BG, other), but not insulin, SGLT2i, SU.

Concomitant antidiabetic medications were allowed throughout the study, with approximately two-thirds of patients receiving additional glucose-lowering therapy. Metformin was the most frequently prescribed concomitant agent (45.7%), followed by gliclazide (27.8%), long-acting insulin (11.6%), dapagliflozin (11.0%), and empagliflozin (10.7%).

### 3.3 Efficacy of sitagliptin and sitagliptin/metformin SPC

One of the primary goals of T2D treatment is to achieve optimal glucose levels to prevent the development of diabetic complications. After six months a decrease in HbA1c, FPG, and PPG average values were observed in all patients with data who were treated with sitagliptin or sitagliptin/metformin SPC.

**Figure 3:**
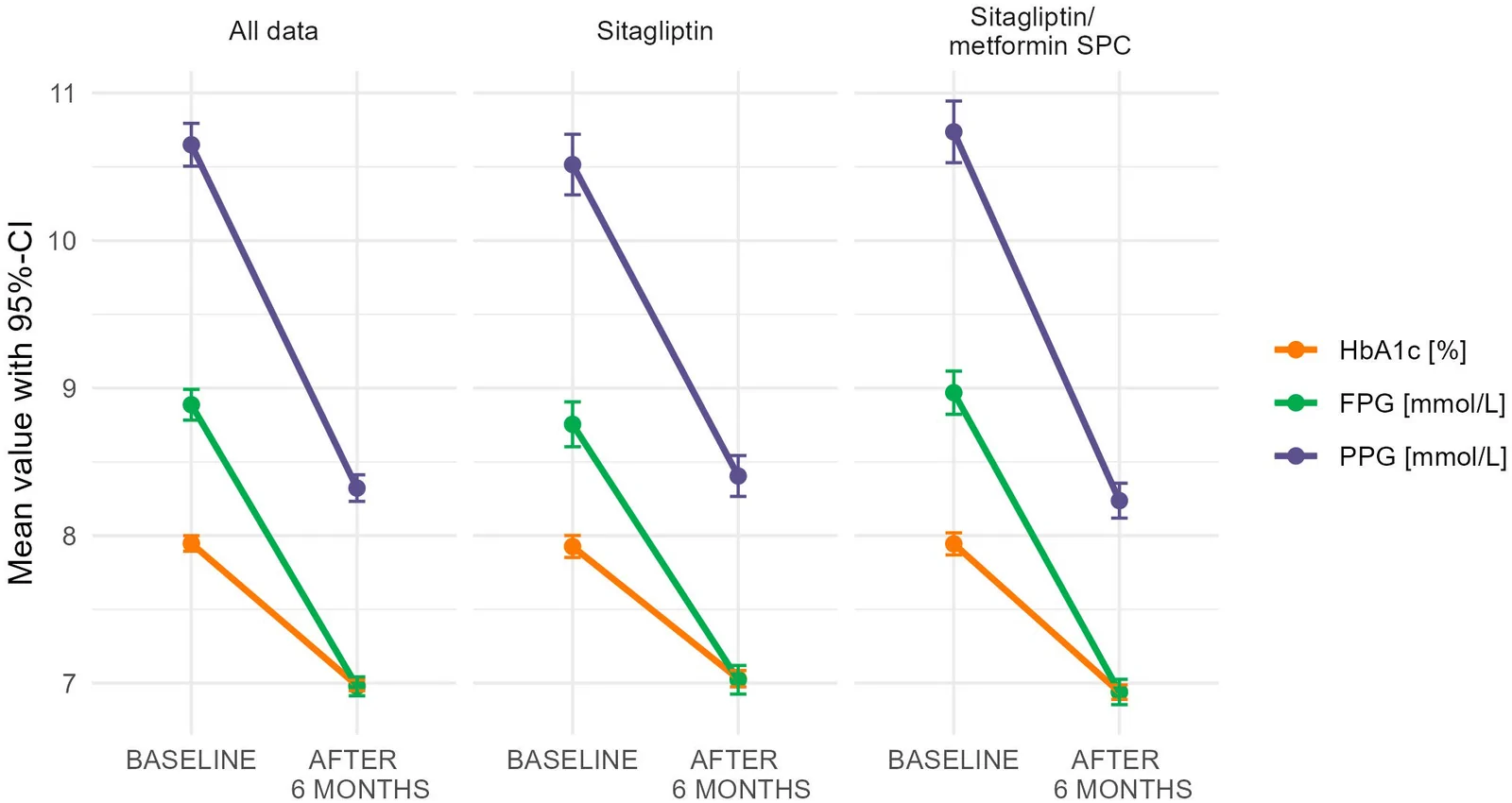
Absolute change in HbA1c, FPG and PPG from baseline to after 6 months (± 1 month).

For 2,065 patients, mean HbA1c decreased from 7.98% at baseline to 6.99% at the third data capture (mean change: −0.99%, 95% CI: −1.05 to −0.93), presented in **Figure 3**. Group 1 and 2 showed consistent HbA1c reduction, results with subgroup analyses (2a–d) are presented in **Table 3**. Group 3 exhibited the largest decrease, from baseline to 6 months (−1.50%, 95% CI: −2.27 to −0.73). All reductions were statistically significant (p < 0.001). A multivariable linear regression model was used to evaluate factors associated with change in HbA1c from baseline to the third data capture. The model included baseline HbA1c, therapy group, therapy changes during follow-up, age, comorbidities, concomitant medication use, hypertension, dyslipidemia, insulin use, and SGLT2 inhibitor use as covariates. The analysis included 2,063 patients with complete data available for all variables entered into the model. The model was statistically significant (F(10, 2052) = 326.2, p < 0.001) and accounted for 61.2% of the variability in HbA1c change (adjusted R² = 0.612). The adjusted mean HbA1c change was −0.77 percentage points (95% CI −0.89 to −0.64). Higher baseline HbA1c was associated with greater HbA1c reduction (β = −0.760, p < 0.001), whereas older age (β = 0.005, p = 0.003), insulin use (β = 0.210, p < 0.001), and SGLT2 inhibitor use (β = 0.192, p < 0.001) were associated with smaller HbA1c reductions. Treatment with sitagliptin/metformin SPC was associated with a modestly greater HbA1c reduction compared with sitagliptin monotherapy (β = −0.085, p = 0.016). Therapy changes during follow-up, comorbidity status, hypertension, dyslipidemia, and concomitant medication use were not significantly associated with HbA1c change. Detailed regression results are provided in **Supplementary Table 1**.

**Table 3:** Absolute changes in HbA1c, FPG and PPG from baseline to after 6 months for patients in groups 1, 2 and subgroups 2a-d.

Group	No. of patients with data	Mean 1st Data Collection	Mean 3rd Data Collection	Mean absolute change	Lower CI	Upper CI	p-value
HbA1c							
Group 1	964	7.97	7.04	-0.93	-1.01	-0.85	<0.001
Group 2	1063	7.97	6.94	-1.03	-1.11	-0.96	<0.001
2a	609	7.68	6.73	-0.95	-1.03	-0.86	<0.001
2b	229	8.38	7.19	-1.19	-1.39	-1.00	<0.001
2c	114	7.88	7.14	-0.74	-0.94	-0.54	<0.001
2d	111	8.83	7.35	-1.48	-1.79	-1.17	<0.001
FPG							
Group 1	867	8.71	7.04	-1.68	-1.85	-1.50	<0.001
Group 2	1015	9.01	6.92	-2.09	-2.25	-1.92	<0.001
2a	592	8.97	6.79	-2.17	-2.38	-1.97	<0.001
2b	214	9.38	7.12	-2.27	-2.68	-1.85	<0.001
2c	103	8.42	7.15	-1.27	-1.67	-0.87	<0.001
2d	106	9.08	7.04	-2.05	-2.65	-1.44	<0.001
PPG							
Group 1	629	10.49	8.36	-2.13	-2.38	-1.88	<0.001
Group 2	751	10.85	8.25	-2.60	-2.85	-2.35	<0.001
2a	412	10.48	8.03	-2.45	-2.79	-2.12	<0.001
2b	183	11.45	8.50	-2.95	-3.53	-2.38	<0.001
2c	75	10.57	8.38	-2.18	-2.76	-1.61	<0.001
2d	81	11.59	8.68	-2.91	-3.65	-2.16	<0.001

Mean FPG decreased after 6 months of treatment with sitagliptin or sitagliptin/metformin SPC. Among 1,917 patients, values declined from 8.89 mmol/L at baseline to 6.98 mmol/L (mean change: −1.91 mmol/L, 95% CI: −2.03 to −1.79) presented in **Figure 3**. Group 1 and 2 showed consistent FPG reduction, results with subgroup analyses (2a–d) are presented in **Table 3**. Group 3 (n = 24) demonstrated the largest absolute decrease (−3.06 mmol/L, 95% CI: −4.60 to −1.53), from 10.09 to 7.02 mmol/L. All reductions were statistically significant (p < 0.001).

PPG values were recorded for 1,401 patients, decreasing from 10.69 mmol/L at baseline to 8.30 mmol/L (mean change: −2.39 mmol/L, 95% CI: −2.56 to −2.21), presented in **Figure 3**. Group 1 and 2 showed consistent PPG reduction, results with subgroup analyses (2a–d) are presented in **Table 3**. All reductions were statistically significant (p < 0.001). Despite the small sample size (n = 14), group 3 exhibited the largest reduction in PPG, with a mean absolute change of −3.07 mmol/L (from 11.57 to 8.50 mmol/L; 95% CI: −5.25 to −0.89), although this change did not reach statistical significance and should be interpreted with caution given the small number of patients with paired PPG data.

At baseline, only 16.9% of 2,442 patients had HbA1c <7% (group 1: 16.6%, group 2: 17.2%, group 3: 18.5%). After 3 months, this proportion increased to 43.0% (group 1: 41.5%, group 2: 45.0%, group 3: 30.8%). At 6 months, more than half of overall patients (53.7%) achieved the target HbA1c <7% (group 1: 51.8%, group 2: 55.6%, group 3: 48.2%).

In the overall population (n = 2,513), mean BMI decreased by −0.61 ± 1.33 kg/m², corresponding to a relative change of −0.02 ± 0.04%. Subgroup results were similar: group 1 (n = 1,152): −0.59 kg/m²; group 2 (n = 1,318): −0.63 kg/m²; group 3 (n = 28): −0.68 kg/m². For an individual 1.7 m tall, this reduction equates to approximately 2 kg, indicating a clinically negligible change.

Across the study population (n = 2,513), BMI remained stable (≤2 kg/m² change) in 87.9% of patients (n = 2,210) between baseline and the third data capture during treatment with sitagliptin or sitagliptin/metformin SPC. Among patients aged ≥65 years (n = 1,293), BMI remained stable in 89.9% (n = 1,163). By subgroup, changes occurred in 9.9% of group 1 (n = 60/606), 10.2% of group 2 (n = 68/667), and 7.7% of group 3 (n = 1/13).

### 3.4 Safety of sitagliptin and sitagliptin/metformin SPC

A total of 49 patients reported adverse reactions (ARs) related to the study medication. Among them, 3 patients reported hypoglycemia related to the study medication (in one patient blood glucose was measured – 3.2 mmol/l, single episode; one patient reported intermittent hypoglycemia, no blood glucose measurements were available, the patient discontinued the medication on their own; one patient reported a single episode accompanied by nausea, with no glucose measurement available). All three events were reported by the treating investigator; none met the criteria for severe hypoglycemia (an episode requiring third-party assistance), none led to hospitalisation, and blood glucose confirmation was available in one case only. No episodes of severe hypoglycemia were reported during the study. This represents 6.1% of all patients with at least one adverse reaction and 0.1% of all patients who were included in the SAS (n=2697).

From 49 patients (1.8%) with AR, 21 patients (0.8%) were treated with sitagliptin and 28 patients (1.0%) with sitagliptin/metformin SPC. Only one patient experienced serious adverse reaction (acute pancreatitis recovered after the treatment discontinuation). Most frequent adverse reactions were mild to moderate upper abdominal pain (n = 16; 0.6%), nausea (n = 9; 0.3%), vomiting (n = 9; 0.3%), diarrhea (n = 7; 0.3%), constipation (n = 6; 0.2%), flatulence (n = 6; 0.2%), dizziness (n = 3; 0.1%) and pruritus (n = 3; 0.1%). 35 patients discontinued the treatment due to AR (1.3%).

### 3.5 Evaluation of reasons for discontinuing previous antidiabetic treatment

At the baseline visit, a total of 1,897 reasons for discontinuation of previous antidiabetic therapy were reported. The most common reason was failure to achieve glycemic targets (55.6%, n = 1,055). Other reasons accounted for 21.8% of cases (n = 413), while 12.4% of patients (n = 236) discontinued treatment due to unaffordable costs. Safety concerns were reported in 10.1% of cases (n = 191), and 9.7% (n = 184) cited inconvenient dosing or mode of administration. In 3.7% of cases (n=71) contraindications of medicine(s) were reported as the reason for discontinuation.

### 3.6 Evaluation of patient’s and investigator’s general satisfaction and patient’s response to sitagliptin and sitagliptin/metformin SPC therapy after 6 months

At the third data capture, feedback on sitagliptin or sitagliptin/metformin SPC therapy was available from 2,512 patients. Overall, 58.2% (n = 1,461) were very satisfied and 32.7% (n = 822) were satisfied. Only 7.2% (n = 181) reported being neutral, while fewer than 2% were dissatisfied (0.7%, n = 17) or very dissatisfied (0.9%, n = 22). Satisfaction data were missing for 9 patients.

Investigator-reported satisfaction was similarly high. Among investigators, 57.8% (n = 1,450) were very satisfied and 33.5% (n = 840) were satisfied. Neutral ratings accounted for 7.3% (n = 182), while 1.0% (n = 26) were dissatisfied and 0.1% (n = 3) very dissatisfied. Data were unavailable for 10 investigators.

Regarding perceived treatment response (n = 2,512), 39.7% (n = 998) of patients rated their condition as very much improved, and 37.4% (n = 939) as much improved. Minimal improvement was reported by 12.0% (n = 302), and no change by 6.6% (n = 166). Only 2.5% (n = 63) assessed themselves as minimally worse, and very few as much worse (n = 7) or very much worse (n = 3). Response data were missing for 34 patients. Furthermore, more than 96% of patients remained on sitagliptin-based therapy throughout the study period.

## 4 Discussion

The non-interventional DIVERSITY study evaluated real-world treatment outcomes of sitagliptin and sitagliptin/metformin single-pill combination (SPC) in type 2 diabetes (T2D) across seven countries. Of 2603 enrolled patients, analyses of glycemic control focused on those with paired baseline and 6-month data for HbA1c, fasting plasma glucose (FPG), and postprandial glucose (PPG). In this population, treatment with sitagliptin or sitagliptin/metformin SPC was associated with improvements in glycemic parameters and was generally well tolerated across diverse T2D subgroups.

In 2065 patients with paired HbA1c data, mean HbA1c declined by 0.99% (95% CI: −1.05 to −0.93) after 6 months, with comparable reductions of 0.93% for sitagliptin monotherapy and 1.03% for sitagliptin/metformin SPC. These changes align with randomized controlled trial (RCT), where sitagliptin monotherapy reduced HbA1c by 0.5% and combination therapy up to 1.5% (9–11). Furthermore, multivariable analysis confirmed a significant reduction in HbA1c after adjustment for baseline characteristics and treatment-related factors, with an adjusted mean HbA1c reduction of 0.77%, suggesting that the association between sitagliptin-based therapy and improved glycemic control remained consistent after accounting for potential confounders. As expected, higher baseline HbA1c was the strongest predictor of HbA1c reduction in the adjusted analysis. Older age, insulin use, and SGLT2 inhibitor use were associated with smaller HbA1c reductions, while treatment with sitagliptin/metformin SPC was associated with a modestly greater reduction compared with sitagliptin monotherapy. These associations may reflect differences in baseline disease severity and treatment complexity among patient subgroups.

Achieving HbA1c <7% is a key target for reducing or preventing T2D microvascular and macrovascular complications (5, 12, 13). At baseline (n = 2442), only 16.9% of patients met this treatment target; after 6 months (n = 2162) this increased to 53.7%, reflecting improved glycemic target attainment over the 6-month observation period.

FPG improved in 1917 patients with paired data (absolute reduction: 1.91 mmol/L; relative: 13.38%), and PPG in 1401 patients (absolute: 2.39 mmol/L; relative: 10.53%), consistent with prior real-world and RCT evidence (11, 14, 15). These improvements in glycemic control, which can lead to diabetes complication risk reduction (16).Weight neutrality is critical in elderly and lean patients, where inadvertent loss may exacerbate frailty or sarcopenia (17). BMI remained stable over 6 months with both regimens.

It is well established that multimorbidity prevalence escalates with age (18) and frequently clusters T2D with hypertension and dyslipidemia (19). In the DIVERSITY study, 87.6% of patients had ≥1 comorbidity, most commonly hypertension (82.1%) and dyslipidemia (77%). Reported hypoglycemia was uncommon, with only 3 of 2,697 patients (0.1%) reporting hypoglycemia related to the study medication. This observation may be particularly relevant, as advanced age, multimorbidity, and polypharmacy substantially elevate hypoglycemia risk, complicating safe glycemic control in these vulnerable populations (20). These findings align with prior research confirming sitagliptin’s low hypoglycemia incidence, whether monotherapy or combined with metformin (11, 14, 21).

At the 6-month follow-up, both patient and physician satisfaction with treatment was high: more than 90% of patients and investigators rated treatment positively, while 77% of investigators reported patients as being “much improved” or “very much improved.” These findings suggest a favorable perception of treatment effectiveness and tolerability among both patients and physicians (22).

Current international (ADA 2026) guidelines recognize DPP-4 inhibitors such as sitagliptin as a treatment option when a low risk of hypoglycemia and weight neutrality are important considerations (23). In the context of our study population, which consisted predominantly of older adults with multiple comorbidities, these characteristics may be particularly relevant because treatment goals often extend beyond glycemic control alone to include treatment tolerability and treatment burden. In our cohort, sitagliptin-based regimens were associated with improvements in glycemic parameters, while rates of reported hypoglycemia remained low and body weight remained largely unchanged. These findings may be relevant for older and multimorbid patients, in whom avoidance of hypoglycemia and weight loss is often taken into account when selecting glucose-lowering therapy. The observed results are generally consistent with previous studies suggesting benefits of early combination therapy for glycemic control (24–28) and add real-world data on outcomes associated with sitagliptin-based treatment in routine clinical practice.

The DIVERSITY study has several strengths. Its real-world, non-interventional design across seven countries (Croatia, Bosnia and Herzegovina, Bulgaria, Hungary, North Macedonia, Romania, and Slovenia) included a broad spectrum of patients with T2D, including elderly (mean age 64.6 years), multimorbid (87.6% with ≥1 comorbidity), and polypharmacy subgroups, reflecting routine clinical practice more closely than randomized controlled trials. The large sample size (n=2603 enrolled; >2000 patients with paired glycemic data) enabled evaluation of glycemic outcomes and safety in a diverse patient population. Furthermore, the assessment of multiple clinical parameters, including HbA1c, FPG, PPG, BMI, comorbidity burden, adverse reactions, and treatment satisfaction, allowed a broad evaluation of treatment outcomes relevant to contemporary T2D management, including glycemic control, hypoglycemia, and body weight. A further strength is the inclusion of patients receiving sitagliptin/metformin SPC, providing insight into the use of this treatment approach in routine clinical practice. The findings were generally consistent with previously published evidence from randomized controlled trials and real-world studies (10, 11, 14, 15, 29).

However, several limitations should be considered when interpreting the findings. As this was a non-interventional study, investigators reported only laboratory and clinical parameters assessed as part of routine clinical practice. Consequently, the availability of data varied across outcomes, with paired HbA1c measurements available for 2,065 patients, FPG measurements for 1,917 patients, and PPG measurements for 1,401 patients. To assess the potential for bias due to missing follow-up laboratory values, we compared baseline HbA1c levels between patients with and without available follow-up HbA1c measurements. Patients with available follow-up measurements had modestly higher baseline HbA1c values compared with those without available follow-up data (7.98% vs 7.76%; mean difference 0.22%, 95% CI 0.07–0.36; *p* = 0.003). Although this difference was statistically significant, its magnitude was small and unlikely to be clinically meaningful, indicating that any bias associated with missing follow-up data is likely to be limited.

The 6-month follow-up captures initial treatment responses but does not allow assessment of the durability of glycemic control, long-term safety, or the impact on diabetes-related complications. Moreover, although the multicountry design may support the generalizability of the findings, differences in prescribing patterns, laboratory methods, healthcare systems, and local clinical practice across participating countries may have introduced heterogeneity that was not specifically evaluated in the present analysis. Future studies with longer follow-up and further exploration of potential between-country differences would help strengthen the evidence base.

In conclusion, the DIVERSITY study contributes real-world evidence on the use of sitagliptin and sitagliptin/metformin SPC in a broad T2D population, including large multimorbid and predominantly elderly cohorts. Their use was associated with improvements in glycemic control and a low incidence of reported adverse reactions during the 6-month observation period. Clinically relevant reductions in HbA1c, FPG, and PPG were observed, consistent with findings from randomized controlled trials and previous real-world studies. BMI remained stable throughout the study, in line with the weight-neutral profile previously reported for these therapies. Hypoglycemia related to sitagliptin or sitagliptin/metformin SPC was reported infrequently (0.1%). Other adverse reactions were mild to moderate and infrequent, with 1.8% of patients reporting any adverse reaction. High satisfaction rates among both patients and investigators were also observed throughout the study. Overall, these findings complement existing evidence on the efficacy, safety, and tolerability of sitagliptin and the sitagliptin/metformin SPC in a diverse patient population reflective of routine clinical practice. Future research with longer follow-up and extended evaluation in highly multimorbid elderly groups would further strengthen the evidence base and help refine therapeutic strategies for real-world diabetes care.

## Data Availability

The raw data supporting the conclusions of this article will be made available by the authors, without undue reservation.
