# Supplementary material for: Real-world outcomes of sitagliptin and sitagliptin/metformin single-pill combination treatment in diverse type 2 diabetes populations (DIVERSITY study)
Source: Front Clin Diabetes Healthc. 2026 Aug 26;7:1905393. doi: 10.3389/fcdhc.2026.1905393 (PMC13555902; doi:10.3389/fcdhc.2026.1905393)
Supplement: Supplementary file 1 [file Table1.docx]

# Supplementary Table S1: Multivariable linear regression analysis of factors associated with change in HbA1c from baseline to the third data capture.

| **Covariate** | **β coefficient** | **95% CI** | **p-value** |
| --- | --- | --- | --- |
| Baseline HbA1c (%) | -0.760 | -0.788 to -0.732 | <0.001 |
| Sitagliptin/metformin SPC vs Sitagliptin | -0.085 | -0.154 to -0.016 | 0.016 |
| Therapy change during follow-up (yes) | 0.086 | -0.111 to 0.284 | 0.391 |
| Age (years) | 0.005 | 0.002 to 0.009 | 0.003 |
| Any comorbidity (yes) | -0.013 | -0.162 to 0.136 | 0.864 |
| Concomitant medication use (yes) | 0.087 | -0.007 to 0.182 | 0.070 |
| Hypertension (yes) | 0.003 | -0.095 to 0.101 | 0.956 |
| Dyslipidemia (yes) | 0.026 | -0.064 to 0.116 | 0.578 |
| Insulin use (yes) | 0.210 | 0.092 to 0.328 | <0.001 |
| SGLT2 inhibitor use (yes) | 0.192 | 0.087 to 0.298 | <0.001 |

HbA1c - glycated hemoglobin; SPC - single-pill combination; SGLT2 - sodium-glucose cotransporter-2.

Multivariable linear regression model adjusted for baseline HbA1c, therapy group, therapy changes during follow-up, age, comorbidities, hypertension, dyslipidemia, concomitant medication use, SGLT2 inhibitor use and insulin use. Overall model: F(10,2052)=326.2, p<0.001; adjusted R²=0.612.
